# In vitro antimicrobial activity of Thai stick cannabis Hang Kra Rog Phu Phan (*Cannabis sativa* L.), sugar leaves extract against pathogenic bacteria

**DOI:** 10.5455/javar.2025.l870

**Published:** 2025-03-24

**Authors:** Panicha Pongnaratorn, Natthida Sophon, Parichart Boueroy

**Affiliations:** 1 Thai Traditional Medicine, Faculty of Natural Resources, Rajamangala University of Technology Isan, Sakon Nakhon Campus, Sakon Nakhon, Thailand; 2 Faculty of Public Health, Kasetsart University Chalermphrakiat Sakon Nakhon Province Campus, Sakon Nakhon, Thailand

**Keywords:** *Cannabis sativa* L., Δ-9- tetrahydrocannabinol, cannabidiol, antimicrobial activity, tannic acid, gallic acid

## Abstract

**Objective:**

*Cannabis sativa* L. is aware of a rich source of bioactive substances with various structures that exhibit pharmacological activity in the central nervous system, cardiovascular, cerebrovascular, respiratory, reproductive, and gastrointestinal systems.

**Materials and Methods:**

In this study, cannabis sugar leaves were soaked in 99% ethanol, followed by evaporation. The antibacterial effect of the cannabis sugar leaf extract was then evaluated using the disc diffusion method. The minimum inhibitory concentration (MIC) and the minimum bactericidal concentration (MBC) were determined using broth dilution.

**Results:**

The results of this study indicated that the cannabis sugar leaf extract inhibited *Bacillus cereus*, *Vibrio cholerae*, *Escherichia coli*, *Staphylococcus aureus*, and *Staphylococcus epidermidis* when compared to tetracycline, but it did not inhibit *Pseudomonas* *aeruginosa*. The MIC and MBC of the cannabis sugar leaves extract against *B*.* cereus*, *V*.* cholerae*, *E*.* coli*, *S*.* aureus*, and *S*.* epidermidis* were 0.977, 1.953, 31.25, 62.5, 125, 250, 250, 500, 250, and 500 mg/ml, respectively. The bioactive compounds in cannabis sugar leaf extract were identified using high-performance liquid chromatography.

**Conclusion:**

The results indicated that the major bioactive compounds were Δ-9- tetrahydrocannabinol (THC) and cannabidiol (CBD). While minor bioactive compounds included gallic acid and tannic acid. These results support the benefits of cannabis sugar leaf extract, which has been used for its pharmacological properties and may be useful as an alternative antimicrobial agent in medicine.

## Introduction

Cannabis, a unique member of the plant kingdom Cannabaceae, belongs to a single genus (*Cannabis*) with one species (*Sativa*), which has many varieties [1]. *Cannabis sativa *L. is characterized by producing fewer flowers, having branches that are farther apart, and a coloration that tends more toward spring green, narrower leaflets, with the plants typically growing taller [2]. This plant contains a great specification of cannabinoids that are not found in any other plant. Moreover, it exhibits a variety of pharmacological properties, including treatments for epilepsy, pain, spasticity, irritable bowel syndrome, and other disorders [1]. Cannabis leaf extract contains over 421 phytochemicals [3]. Many of these compounds are pharmacologically active [3]. Δ-9- tetrahydrocannabinol (THC) and cannabidiol (CBD) are the major cannabinoids found in cannabis [3]. However, CBD’s effects are usually blocked by THC, which acts as an antagonist at cannabinoid receptors, thereby preventing CBD from producing any psychoactive responses. THC is associated with a wide range of psychoactive effects that include feeling “high,” cognitive deficits, perceptual alterations, anxiety, paranoia, deficits in verbal recall in healthy individuals, and the exacerbation of psychotic symptoms in patients with schizophrenia [4]. Numerous psychoactive effects are linked to THC, including feeling “high,” cognitive deficits, perceptual alterations, anxiety, paranoia, deficits in verbal recall in healthy individuals, and the exacerbation of psychotic symptoms in patients with schizophrenia [4].

Moreover, these phytochemicals contain different types of chemicals, including amino acids, nitrogenous compounds, terpenes, simple fatty acids, sugar, and hydrocarbons [5]. Plant secondary metabolites predominantly consist of phenolic compounds, which are commonly found in dietary supplements, beverages, vegetables, chocolate, and fruits. Phenolic compounds are classified into three groups, which include phenolic acids, flavonoids, and tannins. Phenolic acids contain one carboxylic acid functional group and various hydroxylated derivatives of benzoic, such as gallic, protocatechuic, and p-hydroxybenzoic acids [6]. Tannins, which are phenolic compounds soluble in water with a molecular weight between 500 and 3,000 Daltons, are categorized into two types: condensed and hydrolyzable tannins. Gallotannins and ellagitannins are types of hydrolyzable tannins, which can be hydrolyzed by enzymes, acids, or bases into glucose and gallic acid. However, ellagitannins contain various hydroxyphenyl residues, which are linked to glucose as a diester in addition to gallic acid [7]. Tannic acid binds more strongly with iron compared to gallic acid, propyl gallate, or methyl gallate. Tannic acid has an inhibitory effect on the growth of intestinal bacteria due to its strong binding with iron, whereas the mechanisms of propyl gallate and methyl gallate may differ [7].

The predominant antimicrobial targets include physicochemical surface properties, surface charge, intracellular potassium release, and membrane permeabilization [6]. For example, resistance mechanisms include 1) tetracyclines blocking the ribosome by binding to the active site, which alters the target, and 2) energy-dependent efflux of antibiotics (membrane efflux proteins) [8]. The Tet proteins from the major facilitator superfamily are membrane-associated proteins that mediate this efflux system [8]. The export of proteins functions because of their ability to translocate a complex of tetracycline and a divalent metal ion. The positively charged metal ions, such as CO²⁺, support transport, whereas Mg²⁺, Mn²⁺, Cd²⁺, and Ca²⁺ can bind with tetracycline to form a substrate.

Cells are protected from the action of the tetracycline by exporting tetracycline out of the cell [8]. The best-studied determinants of ribosomal protection proteins are Tet (M) and Tet (O), which protect ribosomes when tetracycline binds to the ribosome, preventing the elongation of protein synthesis. These proteins interact with the ribosome, enabling bacterial resistance to tetracycline and supporting cellular growth [8]. The main types of resistance mediation are the protection of ribosomal targets and efflux pumps. Finally, the third mechanism involves cytoplasmic enzymes, which can inhibit tetracycline. Tet (X) is a flavoprotein monooxygenase that can inactivate tetracycline antibiotics by monohydroxylation coupled with spontaneously causing non-enzymatic breakdown [8].

This study aims to identify a new natural antimicrobial source and confirm the major bioactive compounds in cannabis leaf extract. Fresh cannabis leaves were ground, soaked in 99% ethanol, and then evaporated. The bioactive compounds were identified using high-performance liquid chromatography (HPLC). The antimicrobial activity was evaluated using the disc diffusion method, with minimum inhibitory concentration (MIC) and minimum bactericidal concentration (MBC) assessment for *Bacillus cereus, Escherichia coli, Pseudomonas aeruginosa, Staphylococcus aureus, Staphylococcus epidermidis, Vibrio cholerae, *and* Vibrio parahaemolyticus*.

## Materials and Methods

### Cannabis sugar leaves extraction

Cannabis sugar leaves were washed and air-dried. After that, 600 gm of cannabis leaves were ground together with 1.5 l of 99% ethanol and soaked for a week. The supernatant was collected using Whatman No. 1 filter paper. The solvent was removed by evaporation at 45°C. The sample was kept in an amber reagent bottle at –20°C.

### HPLC

For this study, HPLC was conducted using a reverse-phase column. The mobile phase consisted of 90% acetonitrile with 50 mM ammonium formate at pH 3.75 in 10% acetonitrile. The flow rate of the mobile phase under gradient conditions was 1 ml/min for 40 min. Moreover, tannic acid and gallic acid were evaluated using HPLC with a reverse-phase column, where 20% acetonitrile and 80% methanol were used as mobile phases. The flow rate of the mobile phase under gradient conditions was 1 ml/min for 40 min.

### Inoculation and maintenance of microorganisms

Nutrient agar for culturing microorganisms was prepared according to a previous study. To make the nutrient agar, 16.5 gm of agar, 5 gm of peptone, and 3 gm of yeast extract were dissolved in 1 l of distilled water. *Bacillus cereus *(ATCC 11778)*, E*.* coli* (ATCC 25922)*, P*.* aeruginosa *(ATCC 27853)*, S*.* aureus *(ATCC 10832)*, S*.* epidermidis *(ATCC 12228)*, V*.* cholerae*, and* V*.* parahaemolyticus* (ATCC 17802) were inoculated onto nutrient agar at 37°C for 24 h. After that, they were transferred to a nutrient broth and incubated at 37°C for 24 h. They were then transferred to a fresh nutrient broth and incubated at 37°C for a further 4 h. The bacterial cultures were diluted to a concentration of 1.5 × 108 CFU/ml for use in evaluating the antimicrobial activity of cannabis sugar leaf extract.

### Evaluation of the antimicrobial activity of Cannabis sugar leaves

#### Disc diffusion method

The disc diffusion method for antimicrobial susceptibility testing was carried out according to the standard method by Bauer et al. [29] to assess the presence of antibacterial activity in the plant extracts. Bacterial cultures, adjusted to the 0.5 McFarland standard, were evenly spread onto nutrient agar plates using a sterile swab. The plates were dried for 15 min and then used for the sensitivity test. Discs impregnated with plant extracts of 5 mg were placed on the surface of the nutrient agar. Each test plate comprised three discs: one positive control (a standard commercial antibiotic disc), one negative control, and one treated disc. For the treated disc, 10 µl of 500 mg/ml cannabis sugar leaf extract was added and air-dried until the disc was completely dry. The standard antibiotic disc was tetracycline (30 μg) for *B*. *cereus, E*.* coli, P*.* aeruginosa, S*.* aureus, S*.* epidermidis, V*.* cholerae, *and *V*.* parahaemolyticus*. The negative control was a disc with 10 µl of 99% ethanol, which was air-dried. Besides the control discs, each plate had three treated discs placed on it. The plates were then incubated at 37°C for 18 to 24 h, depending on the species of bacteria being tested. After incubation, the plates were examined for inhibition zone. The inhibition zones were then measured using calipers and recorded. The tests were repeated three times to ensure reliability.

#### Broth microdilution method

Broth microdilution method: 100 μl of *B*.* cereus*, *V*.* cholarae*, *E*.* coli*, *S*.* aureus*, and *S*.* epidermidis* were grown in a suitable medium and added to the wells of microtubes already containing 100 μl of two-fold serially diluted plant extract mixed with culture media. The final volume in each tube was 200 μl. Control wells used culture media only, bacterial suspension only, crude extracts prepared with 10-fold serial dilutions (100 µl of 500 mg/ml to 0.977 mg/ml), and ethanol in amounts consistent with the most quantity present. The capacity of each tube was fused at 200 rpm on a shaker for 1 min before incubation for 24 h under the cultivation conditions explained above. The MIC was determined after 24 h, as the lowest concentration was observed based on the metabolic activity where no bacterial viability. After that, each tube was evaluated for the MBC by re-streaking onto nutrient agar and incubating at 37°C for 24 h.

## Results and Discussion

### Cannabis sugar leaves extraction

**Figure 1.**
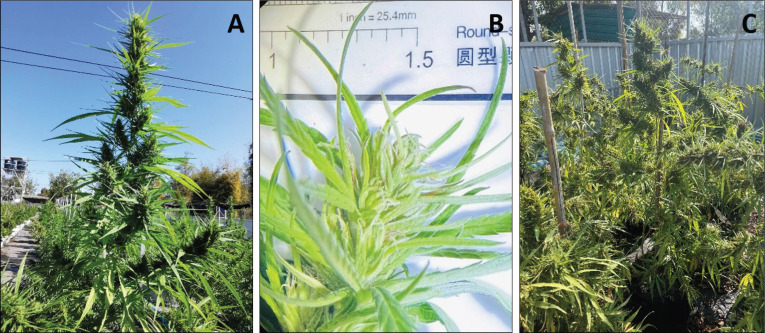
The morphology of Thai stick cannabis Hang Kra Rog Phu Phan. (A) *Cannabis sativa* samples identified as the Hang Kra Rog Phu Phan cultivar, cultivated outdoors at a field demonstration, at the Faculty of Natural Resources, Rajamangala University of Technology Isan, Sakonnakhon Campus. (B) *Cannabis sativa* inflorescence. (C) Cannabis sugar leaves.

Thai stick cannabis, also known as Hang Kra Rog Phu Phan, was used in this research and was identified by Assist. Prof. Ratree Pranakhon from the Faculty of Natural Resources at Rajamangala University of Technology Isan, Sakonnakhon Campus. The cannabis plants were cultivated outdoors at a field demonstration (Fig. 1A). “Thai Stick” is a traditional appearance cigarette that originated in Thailand. It was most popular in the United States during the 1960s and 1970s, as well as during the Vietnam War [9,10]. The cannabis inflorescences were chopped and filled in a bamboo stick, which was then enwrapped in fan leaves and infixed together with hemp string to resemble a cigar [10]. “Hang Kra Rog Phu Phan” is a cannabis strain known for its inflorescences, which resemble the tail of a squirrel. “Phu Phan” is the name of a mountain range in northeastern Thailand, primarily located in Sakon Nakhon Province, with some parts extending into Kalasin Province (Fig. 1B). In this study, sugar leaves were used as samples for extraction (Fig. 1C) to investigate phytochemical compounds using HPLC. Sugar leaves’ Hang Kra Rog Phu Phan were used for the first time for studying. Therefore, this study aimed to fill the gap that there has been no publication exploring Cannabis sugar leaves (Hang Kra Rog Phu Phan) in Thailand. Moreover, previous studies have not been reported on gallic and tannic acid in this plant. This research is the first study to report these acids in it. HPLC’s result is a foundation of knowledge for further study.

These compounds have been the subject of phytochemical investigations for a long time. Moreover, their significant inflammatory and antioxidant properties have been reported [9]. Both THC and CBD are highly lipophilic substances [11], which suggests that these compounds may inhibit microorganism growth by interfering with cell membrane synthesis. A previous study has reported that CBD has an antibacterial ability against *Salmonella typhimurium *and *Salmonella newington* [12]. Furthermore, crude petroleum ether, ethanolic, and aqueous extracts of *C*.* sativa *leaves with isolated acidic fractions were evaluated for antimicrobial activity. The isolated acidic fraction, as well as ethanolic and petroleum ether extracts, exhibited antimicrobial activity against Gram-positive and Gram-negative bacteria and fungi (*Candida albicans* and *Aspergillus niger*), whereas the aqueous extract did not exhibit any activity [13]. In addition, this study investigated tannic and gallic compounds using HPLC.

### Investigation of bioactive compounds of cannabis sugar leaves using HPLC

**Figure 2.**
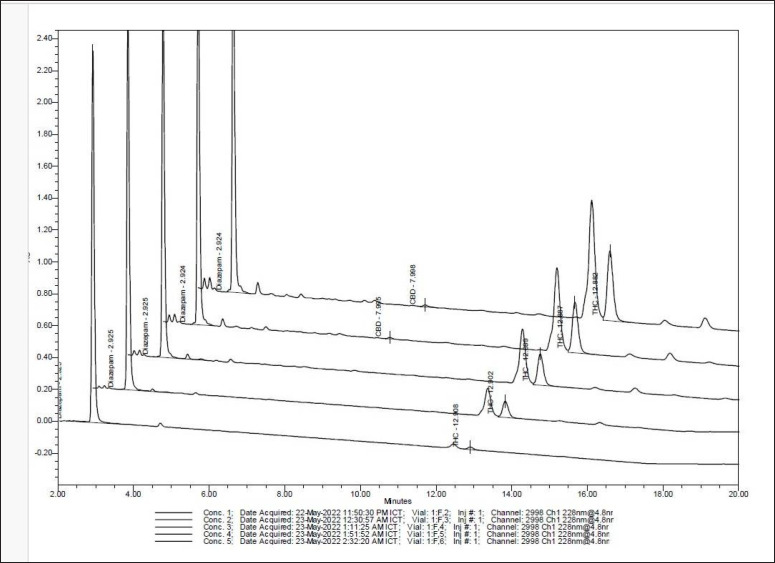
High-performance liquid chromatography (HPLC) results show Δ-9-tetrahydrocannabinol (THC) and cannabidiol (CBD). The HPLC results showed that the cannabis sugar leaves extract contained 2.24 µg/ml of Δ-9-tetrahydrocannabinol (THC) and 2.05 µg/ml of CBD.

The HPLC chromatogram showed that cannabis sugar leaves extract contained 2.24 µg/ml of CBD and 2.05 µg/ml of Δ-9- tetrahydrocannabinol (THC) (Fig. 2). Both are the major pharmacologically active compounds of *C. sativa*. The compounds have been investigated as phytochemical compounds for a long time. Moreover, their antioxidant and anti-inflammatory abilities have been shown [8]. Both THC and CBD are greatly lipophilic substances [9], so these compounds may be microorganism growth inhibitors by cell membrane synthetic inhibition. A previous study reported the antibacterial potential of CBD against *S. newington* and *S*.* typhimurium *[10]. Furthermore, the *C*.* sativa*’s leaves were extracted by petroleum ether, ethanolic, and aqueous extracts, which isolated acidic fractions that were evaluated for antimicrobial activity. Acidic, fracted, ethanolic, and petroleum ether isolation showed antimicrobial activity that inhibits Gram-positive and Gram-negative bacteria and fungi (*C. albicans* and *A. niger*), whereas the aqueous extract did not exhibit any activity [11]. In addition, this study investigated tannic and gallic compounds by HPLC.

The result indicated that cannabis sugar leaf extract contained 0.35 µg/ml of gallic acid and 0.62 µg/ml of tannic acid. Previous research reported a Δ-9-tetrahydrocannabinol (THC) content of 2.05 µg/ml and a CBD content of 2.24 µg/ml (Fig. 2). Moreover, the results indicated that cannabis sugar leaf extract contained tannic and gallic compounds. The antibacterial activity of tannic acid primarily works by inhibiting the NorA efflux pump [14]. This activity is based on the hydroxyl groups in tannic acid forming hydrogen bonds with proteins and other biomolecules. Tannic acid is widely known for its inflammation-reducing and antioxidant properties. Moreover, it also exhibits antibacterial activity against common pathogenic bacteria. Tannins have been reported to have various health effects, such as antinutritional, carcinogenic, mutagenic, anticarcinogenic, and antimutagenic activities [14].

In the next section, the antimicrobial activity of *C*.* sativa *sugar leaves was analyzed by disc diffusion and broth microdilution methods.

### Evaluation of the antimicrobial activity of Cannabis sugar leaves

#### Disc diffusion method

**Figure 3.**
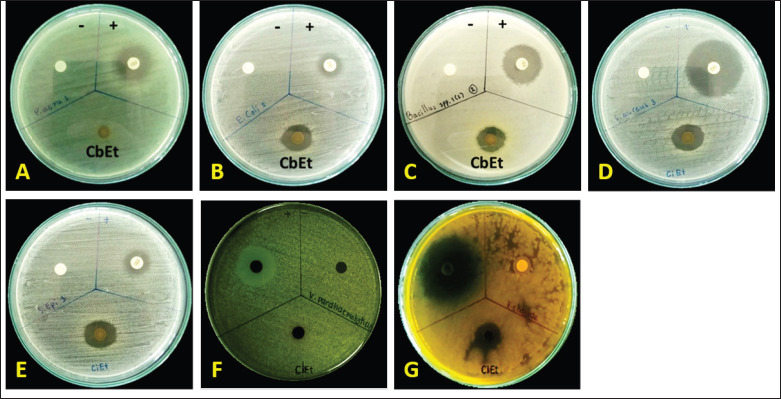
Antimicrobial activity of cannabis sugar leaves extracts on microorganisms. A 500 mg/ml concentration of fresh cannabis sugar leaves extract (CbEt) was loaded onto discs, which were then placed on nutrient agar plates inoculated with each microorganism: (A) *Pseudomonas aeruginosa*, (B) *Escherichia coli, *(C) *Bacillus cereus, *(D) *Staphylococcus aureus, *(E)* Staphylococcus epidermidis, *(F) *Vibrio parahaemolyticus, *and (G) *Vibrio cholerae*. The positive control was 30 µg/ml of tetracycline, and the negative control was 99% ethanol.

The results indicated that disc diffusion experiments revealed that cannabis sugar leaf extract had the greatest antimicrobial effects on *V*.* cholerae *(Fig. 3G), with an inhibition zone of 20.10 mm. This was followed by *S*.* aureus *(Fig. 3D), *S*.* epidermidis *(Fig. 3E), *E*. *coli *(Fig. 3B), and *B*. *cereus *(Fig. 3C), with inhibition zones of 16.4, 15.86, 15.62, and 15.53 mm, respectively. No activity was observed against *P*.* aeruginosa* (Fig. 3A) and *V*.* parahaemolyticus *(Fig. 3F, Table 1). Previous studies reported that cannabis extract showed the greatest antimicrobial effects against *S*.* aureus* 25,923, followed by MRSA, *E*.* coli*, and *K*.* pneumoniae*. However, these extracts did not affect *P*.* aeruginosa* or *A*.* baumannii*. The result of the efficiency of CBCA with microscopic assessment [13] showed that it could induce changes in the bacterial membrane and nucleoid during the development of *B*.* subtilis *[15]. Moreover, the combination of bacitracin and CBD can cause various cell division defects and cell envelope abnormalities [16].

CBD caused a depolarization of the membrane of *S*.* aureus*, disrupting the membrane potential of the bacterium [16]. The loss of genes that regulate the division of cells caused abnormalities in the cells [17]. Another mode of action of cannabinoids is blocking cell communication by inducing the release of bacterial membrane vesicles; however, this effect was not significant in the case of *S*.* aureus* [18]. In addition, studies on bacterial communication systems revealed that HU-210 can interrupt the quorum sensing system’s detection ability and respond to bacterial signals. It can increase the swimming performance of *Vibrio harveyi* [19]. In another study, radiolabeled synthesis tests showed that protein, DNA, and RNA synthesis in *S*.* aureus* RN42200 were significant inhibition of concentrations estimated at the MIC of 2–3 µg/ml [20]. This recommends that rapid bactericidal action depends on shutting down these pathways [21]. Moreover, concentrations below the MIC may reduce lipid synthesis, supporting the hypothesis of membrane-based effects [22].

#### Broth microdilution method

The MIC was analyzed using the broth microdilution method, with microorganisms and nutrient broth serving as positive and negative controls, respectively. The MIC is defined as the lowest concentration of a chemical that inhibits *in vitro* growth of bacteria or fungi [23]. In this study, the MIC results of cannabis sugar leaves extract showed the greatest antimicrobial effects against *B*.* cereus* (0.977 mg/ml), followed by *V*.* cholarae*, *E*.* coli*, *S*.* aureus*, and *S*.* epidermidis,* with MIC values of 31.25, 125, 250, and 250 mg/ml, respectively (Table 2). The MBC is defined as the lowest concentration of an antimicrobial agent that wants to kill a particular bacterium [23]. The MBC measured the lowest concentration of antibacterial agent that decreased the viability of the initial bacterial inoculum with ≥99.9% [24]. In this study, the MBC results of cannabis sugar leaves extract showed the greatest antimicrobial effects against *B*.* cereus* (1.953 mg/ml), followed by *V*.* cholarae*, *E*.* coli*, *S*.* aureus*, and *S*.* epidermidis* with MBC values of 62.5, 250, 500, and 500 mg/ml, respectively (Table 2).

**Table 1.** Antimicrobial activity of cannabis sugar leaves extract.

Microorganisms	Inhibition zone (mm): mean ± SD		
	99% Ethanol (negative control)	Tetracycline (positive control)	Cannabis sugar leaf extract
*Pseudomonas aeruginosa*	-	19.50 ± 0.97	-
*Escherichia coli*	-	13.70 ± 1.05	15.62 ± 2.70
*Bacillus cereus*	-	20.30 ± 0.48	15.53 ± 1.08
*Staphylococcus aureus*	-	31.3 ± 1.25	16.4 ± 0.8
*Staphylococcus epidermidis*	-	13.5 ± 0.52	15.86 ± 1.02
*Vibrio parahaemolyticus*	-	20.10 ± 0.10	-
*Vibrio cholerae*	-	30.17 ± 0.12	20.10 ± 0.10

**Figure 4.**
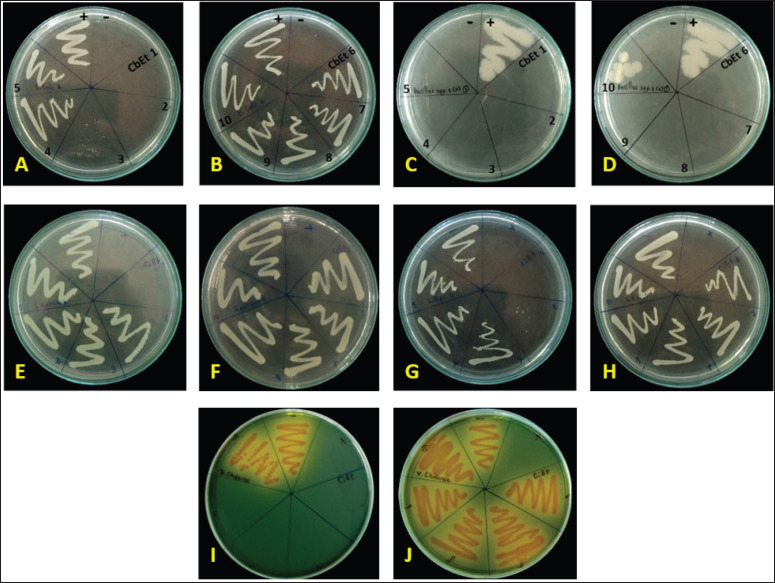
Confirmation of the minimum inhibitory concentration and the minimum bactericidal concentration of Cannabis sugar leaves extract on microorganisms by streak plate method. Different concentrations of cannabis sugar leaves extract were added to inoculated microorganisms, with concentrations ranging from CbEt 1-10; 500, 250, 125, 62.5, 31.25, 15.625, 7.815, 3.906, 1.953, and 0.977 mg/ml, respectively. The samples were then incubated at 37°C for 24 h at 200 rpm on a shaker. Finally, the growth inhibition by cannabis sugar leaf extract was confirmed using a streak plate. (A-B) *Escherichia coli, *(C-D) *Bacillus cereus, *(E-E) *Staphylococcus aureus, *(G-H)* Staphylococcus* *epidermidis, *and (I-J) *Vibrio cholerae*. The positive control was the microorganism itself, while the negative control was the nutrient broth.

The MBC complements the MIC; while the MIC identifies the lowest concentration of an antimicrobial agent that interrupts the growth of bacteria, the MBC indicates the lowest concentration required for an antimicrobial agent to achieve microbial death. The implication of MIC represents inhibition; plating the bacteria onto agar still affects organism proliferation because the antimicrobial did not cause death. Antibacterial agents are generally considered bactericidal agents, while the MBC is not more than four times the MIC [25,26]. Because the MBC test, like a proxy to determine bacterial viability, uses colony-forming units, it can occur due to antibacterial agents that cause aggregation of bacterial cells. Examples of such antibacterial agents include flavonoids [26] and peptides [27,28]. Moreover, these results were confirmed by streak plating, as shown in Figure 4.

**Table 2.** Antimicrobial activity of cannabis sugar leaves extract.

Microorganisms	Concentration of Cannabis sugar leaves extract (mg/ml)	
	MIC	MBC
*Escherichia* *coli*	125	250
*Bacillus cereus*	0.977	1.953
*Staphylococcus aureus*	250	500
*Staphylococcus epidermidis*	250	500
*Vibrio cholerae*	31.25	62.5

## Conclusion

In this study, cannabis sugar leaves are antimicrobial reagents that include *B*.* cereus*, *V*.* cholerae*, *E*.* coli*, *S*.* aureus*, and *S*. *epidermidis* when compared to tetracycline, but they do not inhibit *P. aeruginosa*. The MIC and MBC of the cannabis sugar leaves extract against *B*.* cereus *(0.977 and 1.953 mg/ml), *V*.* cholerae *(31.25 and 62.5 mg/ml), *E*.* coli *(125 and 250 mg/ml), *S*.* aureus *(250 and 500 mg/ml), and *S*.* epidermidis *(250 and 500 mg/ml), respectively. The bioactive compounds in cannabis sugar leaf extract, which are the major bioactive compounds, are Δ-9- tetrahydrocannabinol (THC) and CBD, while minor bioactive compounds include gallic acid and tannic acid. It has been used for its pharmacological properties and may be useful as an alternative antimicrobial agent in medicine. These results back up the benefits of cannabis sugar leaf extract.

## List of abbreviations

C, Celsius; CBCA, Cannabichromenic acid; CBD, cannabidiol; gm, gram; h, hours; HPLC, high-performance liquid chromatography; L, Liter; MBC, the minimum bactericidal concentration; MFS, major facilitator superfamily; mg, milligram; MIC, the minimum inhibitory concentration; ml, milliliter; rpm, rotation per minute; THC, Δ-9- tetrahydrocannabinol; μg, microgram; µl, microliter.
